# Relation between *in Utero* Arsenic Exposure and Birth Outcomes in a Cohort of Mothers and Their Newborns from New Hampshire

**DOI:** 10.1289/ehp.1510065

**Published:** 2016-03-08

**Authors:** Diane Gilbert-Diamond, Jennifer A. Emond, Emily R. Baker, Susan A. Korrick, Margaret R. Karagas

**Affiliations:** 1 Department of Epidemiology, Geisel School of Medicine at Dartmouth, Hanover, New Hampshire, USA; 2 Children’s Environmental Health and Disease Prevention Center at Dartmouth, Hanover, New Hampshire, USA; 3 Department of Biomedical Data Science, Geisel School of Medicine at Dartmouth, Hanover, New Hampshire, USA; 4 Department of Obstetrics and Gynecology, Dartmouth-Hitchcock Medical Center, Lebanon, New Hampshire, USA; 5 Channing Division of Network Medicine, Department of Medicine, Brigham and Women’s Hospital, Harvard Medical School, Boston, Massachusetts, USA; 6 Department of Environmental Health, Harvard T.H. Chan School of Public Health, Boston, Massachusetts, USA

## Abstract

**Background:**

Studies suggest that arsenic exposure influences birth outcomes; however, findings are mixed.

**Objective:**

We assessed in utero arsenic exposure in relation to birth outcomes and whether maternal prepregnancy weight and infant sex modified the associations.

**Methods:**

Among 706 mother–infant pairs exposed to low levels of arsenic through drinking water and diet, we assessed in utero arsenic exposure using maternal second-trimester urinary arsenic, maternal prepregnancy weight through self-report, and birth outcomes from medical records.

**Results:**

Median (interquartile range) of total urinary arsenic [tAs; inorganic arsenic (iAs) + monomethylarsonic acid (MMA) + dimethylarsinic acid (DMA)] was 3.4 μg/L (1.7–6.0). In adjusted linear models, each doubling of tAs was associated with a 0.10-cm decrease (95% CI: –0.19, –0.01) in head circumference. Results were similar for MMA and DMA. Ln(tAs) and ln(DMA) were positively associated with birth length in infant males only; among males, each doubling of tAs was associated with a 0.28-cm increase (95% CI: 0.09, 0.46) in birth length (pinteraction = 0.04). Results were similar for DMA. Additionally, arsenic exposure was inversely related to ponderal index, and associations differed by maternal weight. Each ln(tAs) doubling of tAs was associated with a 0.55-kg/m3 lower (95% CI: –0.82, –0.28, p < 0.001) ponderal index for infants of overweight/obese, but not normal-weight, mothers (pinteraction < 0.01). Finally, there was a significant interaction between maternal weight status, infant sex, and arsenic exposure on birth weight (pinteraction = 0.03). In girls born of overweight/obese mothers, each doubling of tAs was associated with a 62.9-g decrease (95% CI: –111.6, –14.2) in birth weight, though the association was null in the other strata.

**Conclusions:**

Low-level arsenic exposure may affect fetal growth, and the associations may be modified by maternal weight status and infant sex.

**Citation:**

Gilbert-Diamond D, Emond JA, Baker ER, Korrick SA, Karagas MR. 2016. Relation between in utero arsenic exposure and birth outcomes in a cohort of mothers and their newborns from New Hampshire. Environ Health Perspect 124:1299–1307; http://dx.doi.org/10.1289/ehp.1510065

## Introduction

Fetal development is a period marked by rapid cell division, differentiation, and tissue growth and is therefore highly sensitive to the negative effects of toxic exposures. Arsenic is a naturally occurring chemical element found in bedrock and soil and it readily dissolves into surrounding groundwater that may be used for drinking (Smedley and Kinniburgh 2002). There is also growing awareness that people are exposed to arsenic through consumption of rice and other foods (Gilbert-Diamond et al. 2011; Meharg et al. 2009). Arsenic crosses the placenta (Concha et al. 1998), and experimental studies suggest that arsenic may have reproductive toxicity (Ahmed et al. 2011; Davey et al. 2007; Tsang et al. 2012), though the epidemiological evidence relating arsenic exposure to fetal growth has been mixed. Three prospective studies have found negative associations between arsenic exposure biomarkers and birth weight (Claus Henn et al. 2016; Laine et al. 2015; Rahman et al. 2009; Yang et al. 2003), and a third found a suggestive negative association (Hopenhayn et al. 2003). In contrast, two large cross-sectional studies did not find any association between arsenic and birth weight (Kwok et al. 2006; Myers et al. 2010). Almost all of these studies were performed in arsenic-endemic regions with generally high water arsenic (e.g., > 50 μg/L), and few studies have examined arsenic–birth outcome relationships in areas with predominantly lower levels of arsenic exposure (e.g., drinking-water arsenic < 10 μg/L).

After ingestion, inorganic arsenic (iAs) is methylated into monomethylarsonic acid (MMA^V^), which is then reduced to monomethylarsonous acid (MMA^III^) (Vahter 2002). In a second methylation step, MMA^III^ is converted to dimethylarsinic acid (DMA^V^), which is then reduced to dimethylarsinous acid (DMA^III^). This biotransformation of iAs is incomplete and iAs, MMA^V^, and DMA^V^ are all excreted in the urine (Francesconi et al. 2002; Watanabe and Hirano 2013). Although the reduced methylated forms, MMA^III^ and DMA^III^, have also been found in urine of people exposed to high levels of iAs (Mandal et al. 2001; Valenzuela et al. 2005), they have not been observed in urine samples from populations exposed to lower levels of arsenic (Lindberg et al. 2006; Rivera-Núñez et al. 2012). MMA^V^ and DMA^V^ are generally considered less toxic than iAs (Gebel 2002; Moore et al. 1997); however, some animal studies suggest that MMA^III^ and DMA^III^ may be even more toxic than iAs (Styblo et al. 2000).

Weight status may affect the efficiency of arsenic metabolism (Gomez-Rubio et al. 2011; Li et al. 2008), although findings have been mixed. For example, a study among 624 adult women in the southern United States and northern Mexico (Gomez-Rubio et al. 2011) where 68.9% of women were overweight or obese, documented that the efficiency of arsenic metabolism increased with maternal body mass index (BMI). In contrast, a study among 442 Bangladeshi pregnant women (Li et al. 2008) documented no statistical differences in arsenic methylation rates by maternal weight status during the first trimester; however, only 5.4% of the women in that sample were overweight (BMI ≥ 25 kg/m^2^). Given the importance of maternal weight status in fetal growth (Hull et al. 2008; King 2006; Sewell et al. 2006; Wu et al. 2004) and maternal weight status’s potential role in arsenic metabolism, it is important to understand if maternal weight status modifies arsenic’s association with fetal growth outcomes.

The aims of this study were to assess the associations between *in utero* arsenic exposure and birth size among a cohort of U.S. pregnant women with low-level arsenic exposure from diet and drinking water, and to determine whether maternal prepregnancy weight modified that association.

## Methods

### Source Population

The New Hampshire Birth Cohort Study (NHBCS) is a prospective study designed to examine the associations of arsenic exposure on fetal growth and development during early childhood (Gilbert-Diamond et al. 2011). In the state of New Hampshire, residents commonly use unregulated private water supplies, and it is estimated that more than 1 in 10 homes have drinking-water wells with water arsenic concentrations that exceed the U.S. Environmental Protection Agency’s recommended maximum of 10 μg/L (Karagas et al. 1998). Pregnant women 18–45 years old between 24 and 28 weeks of gestation were recruited from prenatal clinics in New Hampshire beginning in January 2009. Eligibility criteria included English literacy, the use of a private, unregulated water system (e.g., private well) at home, and a singleton pregnancy. The NHBCS enrolled 1,033 pregnant women by 30 September 2013. At the time of the analysis there were 1,020 live singleton births. Of those, prepregnancy weight status was not available for 111 women, maternal second-trimester spot urine samples were not available for an additional 151 women, and newborn anthropometric data were not available for 27 newborns. Of the remaining 731 mother–child dyads, 25 were excluded because the mother had a prepregnancy BMI < 18.5 kg/m^2^. Thus the final sample size of the analysis was 706 dyads. When comparing women who were included in the analysis with the entire population-based cohort, there were no significant differences related to baseline characteristics, arsenic exposures, or birth outcomes (data not shown). Participants provided informed consent, and all study procedures were approved by the Internal Review Board at Dartmouth College.

### Primary Outcomes: Measures of Infant Size at Birth

Infant length, weight, head circumference, gestational age (weeks), and sex at birth were collected from a medical record review by trained study staff. Ponderal index at birth was computed as kg/m^3^, and age- and sex-adjusted birth lengths and birth weight percentiles were computed per the methods of Fenton (Fenton et al. 2013). Age- and sex-adjusted birth weight percentiles were used to classify infants’ size-for-gestational age, which included small (≤ 10th percentile) (SGA), average (11–89th percentile) (AGA), and large-for-gestational age (≥ 90th percentile) (LGA) (Lesseur et al. 2013; Wilhelm-Benartzi et al. 2012).

### Primary Exposure: *in Utero* Arsenic Exposure

Using previously described methods (Gilbert-Diamond et al. 2011), we collected and analyzed maternal second-trimester urinary arsenic. Women provided a spot urine sample collected at approximately 24–28 weeks of gestation that was analyzed for iAs (As^III^ and As^V^), MMA^V^, and DMA^V^ (hereafter MMA and DMA) using a high-performance liquid chromatography inductively coupled plasma mass spectrometry system (Larsen et al. 1993). The limits of detection (LODs) ranged from 0.01 to 0.39 μg/L for the individual arsenic species across the analysis batches. The average LOD for iAs was 0.15 μg/L, and the average LOD for both MMA and DMA was 0.06 μg/L. Measurements below the detection limit were set to half the LOD. Total urinary arsenic concentrations (tAs) were determined by summing those four metabolites and excluding arsenobetaine, an organoarsenic compound that is unmetabolized and is thought to be nontoxic (Francesconi et al. 2002). Inorganic arsenic, MMA^V^, DMA^V^, and tAs concentrations were right-skewed, so values were natural log (ln)–transformed for analysis. In addition, we divided MMA^V^ by iAs and DMA^V^ by MMA^V^ to calculate the primary and secondary methylation indices (PMI and SMI), respectively (Chen et al. 2003; Gilbert-Diamond et al. 2013; Tseng 2009). The PMI and SMI were analyzed as continuous predictors. Urinary creatinine, a marker of urinary dilution, was measured using a calorimetric assay (Assay #500701; Cayman Chemical, Ann Arbor, MI).

In addition to providing urine samples, women also provided drinking-water samples that were analyzed for arsenic as previously described (Gilbert-Diamond et al. 2011). Because rice (Cascio et al. 2011; Gilbert-Diamond et al. 2011; Meharg et al. 2009) and seafood (Adams et al. 1994; Francesconi 2010) are also known sources of arsenic exposure in the United States, women also reported their rice and seafood intake for the 3 days before the urine sample collection on a brief questionnaire.

### Maternal Weight Status

Women were asked to report their usual weight when not pregnant as part of the enrollment questionnaire. Those values were combined with height abstracted from the medical records to compute prepregnancy BMI (kg/m^2^). Prepregnancy weight status was categorized as normal weight (18.5–25 kg/m^2^), or overweight or obese (≥ 25 kg/m^2^). Twenty-five women who were classified as underweight (< 18.5 kg/m^2^) before the current pregnancy were excluded from this analysis.

### Other Measures

Demographics and lifestyle characteristics and medical history were collected via prenatal and postpartum questionnaires. Trained study staff also conducted a medical record review to collect results of gestational blood glucose testing. As part of routine prenatal care, women typically completed a nonfasting glucose challenge test (GCT) between 24 and 28 weeks gestation, where blood was sampled 1 hr after ingestion of 50 g of glucose. For those with a 1-hr GCT result of 140–199 mg/dL an additional, fasting oral glucose tolerance test (OGTT) was performed, where blood was sampled hourly for 3 hr after ingesting 100 g of glucose. Because women with a 1-hr GCT result ≥ 200 mg/dL were considered to have had gestational diabetes, they did not undergo a subsequent OGTT. For this analysis, results from both the GCT and OGTT were used to define maternal gestational hyperglycemia. Specifically, gestational diabetes mellitus (GDM) was defined as a 1-hr GCT result ≥ 200 mg/dL or failing the 1-hr GCT (140–199 mg/dL) with two or more high values on the OGTT based on the American Diabetes Association criteria for a normal OGTT (Committee on Practice Bulletins—Obstetrics 2013) of blood glucose ≤ 95 mg/dL at baseline, ≤ 180 mg/dL at 1 hr, ≤ 155 mg/dL at 2 hr, and ≤ 140 mg/dL at 3 hr. Impaired glucose tolerance (IGT) was defined as failing the 1-hr GCT (140–199 mg/dL) and having one high value on the OGTT. For two participants, results from 1-hr GCT were not available, yet a diagnosis of GDM was included in the medical record. Therefore, data on IGT status were available for 680 participants, and data on GDM status were available for 682 participants.

### Statistical Analyses

All analyses were completed using R: A Language and Environment for Statistical Computing, version 3.0.2 (R Core Team 2013). A threshold of *p* < 0.05 was used to define associations as statistically significant. Baseline characteristics, maternal urinary arsenic concentrations, infant gestational age, and infant birth size measures were summarized overall and compared by maternal prepregnancy weight status using chi-square test for categorical measures, *t*-tests for normally distributed continuous measures, and Wilcoxon rank-sum tests for non-normally distributed continuous measures.

We assessed the linearity of each dose–response relationship between each arsenic exposure (ln-transformed) and each outcome using generalized additive models (GAMs). GAMs were fit using the R language gam function included in the mgcv package. In our analyses, the shape of dose–repose relationship was estimated using thin plate splines (Wood 2004). The quasi-Akaike Information Criteria (qAIC) is an indicator of model fit, with lower values indicating a better-fitting model (Wood 2004). When considering the inclusion of a nonlinear term in each model, the criterion of a change in qAIC of ≥ 10 (Burnham and Anderson 2002) was considered evidence of an improved model fit over a linear model. Per that criterion, we did not find evidence of any nonlinear dose–response relationships between total arsenic (ln-transformed) and each outcome.

Models were fit in the overall sample and stratified by maternal prepregnancy weight status and infant sex. In all models, variables associated with maternal prepregnancy weight status or birth size at the *p* < 0.10 level in bivariate analyses were included as covariates. In this study, we explored arsenic’s association with birth anthropometric outcomes while controlling for gestational age, and did not explore potential mediation of any associations by gestational age. The models for the head circumference, birth length, birth weight, and ponderal index outcomes were adjusted for maternal age at enrollment (continuous), maternal education (ordinal, with the five categories listed in Table 1 assigned values of 1–5, respectively), maternal parity (continuous), infant sex, and gestational age (continuous). Models for the gestational age outcome were similar except they did not adjust for gestational age. Models for the sex- and age-specific birth length or weight percentiles were also similar but they did not adjust for gestational age or infant sex. Models with PMI and SMI as the main predictors were additionally adjusted for tAs. For each outcome, models for the overall sample also included maternal prepregnancy weight status (dichotomous: BMI < 25 kg/m^2^, BMI ≥ 25 kg/m^2^) as a covariate.

**Table 1:** Selected characteristics of 706 mothers and their infants from a New Hampshire pregnancy cohort for all participants and by maternal prepregnancy weight status.*^a^*

Variable	All (*n* = 706)	Normal weight (*n* = 406)	Overweight/obese (*n* = 300)	*p*-Value^*b*^
Prepregnancy BMI (kg/m^2^) (mean ± SD)	25.3 ± 4.9	22.1 ± 1.6	29.8 ± 4.4	< 0.001
Maternal age (years) (mean ± SD)	31.1 ± 4.7	31.4 ± 4.7	30.6 ± 4.7	0.03
Education level [*n* (%)]^*c*^
Less than 11th grade	8 (1.1)	3 (0.7)	5 (1.7)	0.01
High school graduate or equivalent	68 (9.7)	34 (8.4)	34 (11.4)
Junior college graduate, or some college or technical school	153 (21.8)	77 (19.1)	76 (25.4)
College graduate	275 (39.1)	157 (38.9)	118 (39.5)
Postgraduate schooling	199 (28.3)	133 (32.9)	66 (22.1)
Previous pregnancies [*n* (%)]^*d*^
0	281 (39.9)	172 (42.6)	109 (36.3)	0.04
1	271 (38.5)	151 (37.4)	120 (40.0)
2	96 (13.6)	58 (14.4)	38 (12.7)
≥ 3	56 (8.0)	23 (5.7)	33 (11.0)
Smoking status [*n* (%)]
Never	624 (88.4)	357 (87.9)	267 (89.0)	0.58
Ever^*e*^	45 (6.4)	29 (7.1)	16 (5.3)
Current	37 (5.2)	20 (4.9)	17 (5.7)
Maternal gestational weight gain (lbs) (mean ± SD)^*f*^	32.0 ± 12.1	33.8 ± 9.6	29.5 ± 14.5	< 0.001
Maternal gestational hyperglycemia [*n* (%)]^*g*^
Normal	566 (83.0)	332 (84.7)	234 (80.7)	0.37
IGT	70 (10.3)	37 (9.4)	33 (11.4)
GDM	46 (6.7)	23 (5.9)	23 (7.9)
1-Hr blood glucose challenge test results^*g*^ (mg/dL) (mean ± SD)	113 ± 28	112 ± 27	114 ± 29	0.29
Home water arsenic concentration (μg/L) [median (IQR)]	0.5 (0.1–2.7)	0.5 (0.1–2.6)	0.5 (0.1–2.8)	0.94
Any seafood in 2 days before urine collection [*n* (%)]	112 (15.9)	69 (17.0)	43 (14.3)	0.39
Any rice in 2 days before urine collection [*n* (%)]	163 (23.1)	102 (25.1)	61 (20.3)	0.16
Urinary arsenic concentration (μg/L) [median (IQR)]
Inorganic arsenic (iAs)	0.3 (0.1–0.5)	0.2 (0.1–0.5)	0.3 (0.1–0.5)	0.49
Monomethylarsonic acid (MMA)	0.3 (0.1–0.5)	0.3 (0.1–0.5)	0.3 (0.1–0.6)	0.41
Dimethylarsinic acid (DMA)	2.8 (1.4–4.9)	2.5 (1.3–4.8)	2.9 (1.4–5.1)	0.27
Total Arsenic (iAs + MMA + DMA)	3.4 (1.7–6.0)	3.3 (1.6–5.7)	3.6 (1.8–6.1)	0.34
Primary methylation index (MMA/iAs)	1.0 (0.5–1.8)	1.0 (0.5–1.8)	1.0 (0.5–1.8)	0.84
Secondary methylation index (DMA/MMA)	10.4 (7.6–18.3)	10.3 (7.6–17.7)	10.6 (7.5–18.6)	0.68
Infant delivery mode [*n* (%)]
Vaginal	472 (66.9)	291 (71.7)	181 (60.3)	< 0.01
Cesarean section	234 (33.1)	115 (28.3)	119 (39.7)
Infant birth outcomes
Gestational age (weeks) (mean ± SD)	39.5 ± 1.6	39.6 ± 1.5	39.3 ± 1.7	0.02
Head circumference (cm) (mean ± SD)	34.7 ± 1.7	34.5 ± 1.8	34.9 ± 1.7	< 0.01
Birth length (cm) (mean ± SD)	50.9 ± 2.7	50.9 ± 2.6	50.9 ± 2.8	0.92
Birth weight (g) (mean ± SD)	3,462 ± 516.8	3,435 ± 488.6	3,499 ± 551.5	0.11
Ponderal index (kg/m^3^) (mean ± SD)	26.3 ± 3.4	26.1 ± 3.3	26.6 ± 3.6	0.07
Birth weight status [*n* (%)]^*h*^
Small for gestational age	37 (5.2)	24 (5.9)	13 (4.3)	0.02
Average for gestational age	600 (85.0)	353 (87.0)	247 (82.3)
Large for gestational age	69 (9.8)	29 (7.1)	40 (13.3)
^***a***^Normal weight defined as prepregnancy BMI 18.5–24.9 kg/m^2^ and overweight/obese defined as prepregnancy BMI ≥ 25 kg/m^2^; 25 underweight mothers (BMI < 18.5 kg/m^2^) excluded from analysis. ^***b***^Calculated from an unpaired, 2-tailed *t*-test with an equal variance assumption to analyze the difference in means, a chi-square test to analyze the difference in proportions, or a Wilcoxon–Mann–Whitney test to analyze the difference in medians by maternal weight status. ^***c***^Education data missing for 3 participants. ^***d***^Parity data missing for 2 participants. ^***e***^Participants who reported ever smoking but did not report smoking during pregnancy or had missing data for smoking during pregnancy were classified as ever smokers. ^***f***^Data for gestational weight gain available for a subset of 639 women. ^***g***^Maternal hyperglycemia measured as part of usual prenatal care. Results for 1-hr blood glucose screening and IGT status available for a subset of 680 women; data on GDM status available for a subset of 682 women. ^***h***^Age- and sex-adjusted birth weight percentiles were used to classify infants’ size-for-gestational age, which included small (≤ 10th percentile) (SGA), average (11–89th percentile) (AGA), and large-for-gestational age (≥ 90th percentile) (LGA) (Fenton et al. 2013).				

Models with multiplicative interaction terms between the urinary As exposure and maternal prepregnancy weight status were used to formally assess modification by maternal prepregnancy weight status; a Wald test was used to determine statistical significance of the multiplicative terms. In addition, because other studies report potential sex-specific effects of arsenic exposure (Davis et al. 2015; Kippler et al. 2012; Pilsner et al. 2012), we conducted similar interaction analyses to test for effect modification by infant sex on the relationships between arsenic and birth outcomes. For birth outcomes that showed evidence of effect modification (either statistically significant or suggestive) by either maternal weight status or infant sex, we estimated the associations with each arsenic exposure stratified by both maternal weight status and infant sex. To test for the significance of a three-way interaction between maternal weight status, infant sex, and each arsenic exposure, we fit a multivariable regression with main effect terms, two-way multiplicative interaction terms, and a three-way multiplicative interaction term, adjusted for covariates; we assessed the statistical significance of each three-way multiplicative interaction term using a Wald’s test.

Several sensitivity analyses were completed to assess the robustness of the results. All multivariate regression models were repeated: *a*) excluding women who reported any seafood intake within the 2 days before the urine sample collection, *b*) excluding any women who reported any rice intake within the 2 days before the urine sample collection, and *c*) adjusted for creatinine for the subset of women with urinary creatinine concentrations available. We also repeated the analysis with the head circumference outcome additionally adjusting for *a*) vaginal or cesarean birth and *b*) birth weight. Finally, because prepregnancy weight status is positively associated with risk of developing GDM (Torloni et al. 2009), we completed exploratory analyses to determine if there was a multiplicative interaction between maternal hyperglycemia and arsenic exposure on infant birth size using a Wald test to determine statistical significance of the interaction terms.

## Results

Among the 706 included participants, 300 (42.5%) were overweight or obese before their current pregnancy, and specifically 113 (16.0%) were obese. Table 1 presents baseline characteristics by maternal prepregnancy weight status. Three women were missing education data, 2 women were missing parity data, and 67 women were missing gestational weight gain data. In addition, 25 women were missing data on GDM status, and 27 women were missing data on IGT and their 1-hr blood glucose screening. Women who were overweight or obese were younger, more likely to have a lower level of education, and less likely to be nulliparous. Home water arsenic concentrations and any intake of seafood or rice did not differ significantly by prepregnancy weight status. Rates of IGT and GDM did not differ significantly by prepregnancy weight status, nor did mean screening blood glucose. Urinary iAs was correlated with MMA and DMA (Spearman’s ρ = 0.71, 0.59, respectively; both *p* < 0.001) and tAs was most strongly correlated with DMA (Spearman’s ρ = 0.98, *p* < 0.001). Unadjusted concentrations of maternal urinary arsenic did not differ by prepregnancy weight status, nor did the PMI and SMI.

Infant birth characteristics by maternal prepregnancy weight status are presented in Table 1. Maternal overweight or obese weight status was associated with an earlier gestational age at birth, though there was no difference in the proportion of births before 37 weeks by weight status (5.4% overall, *p* = 0.43). Overweight or obese status was also associated with a larger head circumference at birth. When considering age- and sex-adjusted size at birth (data not shown), the average (SD) birth length percentile was 57 (29) among all infants and did not differ by maternal weight status (*p* = 0.12). The average (SD) birth weight percentile was significantly higher for infants whose mothers were overweight or obese compared to normal weight prepregnancy [57 (27) vs. 51 (26); *p* = 0.001]. Additionally, mothers who were overweight or obese gave birth to children with a 0.5 kg/m^3^ higher ponderal index, on average, though this difference was only marginally statistically significant (*p* = 0.07).

Results from the adjusted generalized additive models supported linear dose–response relationships between maternal urinary arsenic concentrations and gestational age, head circumference, birth length, birth weight, ponderal index, birth length percentile, and birth weight percentile (data not shown). Table 2 presents the adjusted parameter estimates from a series of linear regression models fitting those infant birth characteristics on natural log–transformed maternal urinary arsenic concentrations, overall and stratified by maternal prepregnancy weight status. Maternal urinary MMA and tAs were negatively associated with infant head circumference at birth. After holding all other covariates constant, each doubling of tAs was associated with a 0.10-cm decrease [95% confidence interval (CI): –0.19, –0.01, *p* = 0.03] in head circumference. The association was not modified by maternal weight status and did not change after further adjustment for delivery mode and/or birth weight (see Table S1). Results were also consistent in unadjusted models (data not shown). The negative associations between maternal urinary iAs and DMA and head circumference were similar in magnitude to those for MMA and tAs, though they did not reach statistical significance.

**Table 2:** Adjusted parameter estimates (95% confidence interval) for the change in birth outcomes with each unit increase in natural log–transformed maternal 2nd trimester urinary arsenic for all participants and by maternal prepregnancy weight status (*n* = 706).*^a^* ^,^ *^b^*

Outcome/maternal urinary arsenic variable	Overall (*n *= 706)	Normal weight (*n *= 406)	Overweight/obese (*n *= 300)	*p* for interaction^*c*^
Gestational age (weeks)^*d*^
Ln(iAs)	0.04 (–0.06, 0.15)	0.10 (–0.03, 0.23)	–0.04 (–0.20, 0.12)	0.18
Ln(MMA)	0.05 (–0.04, 0.14)	0.08 (–0.03, 0.20)	0.00 (–0.13, 0.14)	0.38
Ln(DMA)	0.09 (–0.03, 0.21)	0.11 (–0.05, 0.27)	0.07 (–0.11, 0.26)	0.78
Ln(tAs)	0.10 (–0.03, 0.22)	0.12 (–0.05, 0.28)	0.07 (–0.11, 0.26)	0.74
Head circumference (cm)^*e*^
Ln(iAs)	–0.08 (–0.19, 0.02)	–0.12 (–0.26, 0.02)	–0.04 (–0.21, 0.13)	0.51
Ln(MMA)	–0.10 (–0.19, –0.01)*	–0.08 (–0.19, 0.04)	–0.14 (–0.28, 0.00)*	0.49
Ln(DMA)	–0.12 (–0.25, 0.00)	–0.13 (–0.29, 0.04)	–0.11 (–0.30, 0.08)	0.90
Ln(tAs)	–0.14 (–0.27, –0.01)*	–0.15 (–0.32, 0.02)	–0.12 (–0.31, 0.07)	0.83
Birth length (cm)^*e*^
Ln(iAs)	0.06 (–0.10, 0.22)	0.01 (–0.20, 0.22)	0.13 (–0.12, 0.39)	0.46
Ln(MMA)	0.10 (–0.04, 0.24)	0.02 (–0.16, 0.20)	0.21 (0.00, 0.43)	0.18
Ln(DMA)	0.22 (0.03, 0.41)*	0.09 (–0.16, 0.35)	0.38 (0.09, 0.67)*	0.14
Ln(tAs)	0.21 (0.01, 0.40)*	0.07 (–0.19, 0.33)	0.37 (0.08, 0.67)*	0.12
Birth weight (g)^*e*^
Ln(iAs)	–9.7 (–38.4, 19.0)	–0.5 (–37.9, 37.0)	–22.9 (–67.6, 21.8)	0.45
Ln(MMA)	3.9 (–20.5, 28.3)	19.4 (–12.4, 51.3)	–18.0 (–55.8, 19.8)	0.14
Ln(DMA)	4.1 (–29.5, 37.8)	24.0 (–20.5, 68.4)	–21.9 (–72.8, 29.0)	0.18
Ln(tAs)	–1.3 (–35.8, 33.2)	15.8 (–30.1, 61.8)	–23.1 (–74.9, 28.7)	0.27
Ponderal index (kg/m^3^)^*e*^
Ln(iAs)	–0.17 (–0.39, 0.05)	–0.01 (–0.30, 0.28)	–0.40 (–0.75, –0.06)*	0.08
Ln(MMA)	–0.13 (–0.32, 0.06)	0.11 (–0.13, 0.35)	–0.47 (–0.76, –0.19)*	< 0.01
Ln(DMA)	–0.32 (–0.58, –0.06)*	0.04 (–0.30, 0.38)	–0.79 (–1.18, –0.40)**	< 0.01
Ln(tAs)	–0.34 (–0.60, –0.07)*	0.02 (–0.33, 0.37)	–0.79 (–1.18, –0.40)**	< 0.01
^***a***^Normal weight defined as prepregnancy BMI 18.5–24.9 kg/m^2^ and overweight/obese defined as prepregnancy BMI ≥ 25 kg/m^2^; 25 underweight mothers (BMI < 18.5 kg/m^2^) excluded from analysis. ^***b***^Results from a series of regression models fitting each birth outcome on each arsenic exposure measure [natural log–transformed inorganic arsenic (iAs), monomethylarsonic acid (MMA), dimethylarsinic acid (DMA), total arsenic (iAs + MMA + DMA) (tAs)]; models are adjusted as noted. ^***c***^*p*-Value from a two-sided Wald’s *t*-test testing the significance of a multiplicative term of maternal prepregnancy weight status (categorical) and each arsenic exposure in the regression model. ^***d***^Model adjusted for maternal age (continuous), education level (ordinal with the five categories listed in Table 1 assigned values of 1–5, respectively), parity (continuous), and infant sex; models for overall sample additionally adjusted for maternal prepregnancy weight status (categorical). ^***e***^Model adjusted for variables described in footnote *d* as well as gestational age (continuous). *p < 0.05. **p < 0.001.				

Maternal urinary DMA and tAs were positively associated with infant birth length. After holding all other covariates constant, each doubling of tAs was associated with a 0.15-cm increase (95% CI: 0.01, 0.28, *p* = 0.04) in birth length. Although the estimates of association were larger and only statistically significant in the overweight/obese stratum, the statistical test for interaction by maternal weight status did not reach statistical significance. The results for birth length percentile were qualitatively similar to those for birth length (see Table S2); birth length percentile was positively associated with ln(DMA) and ln(tAs) in the stratum of overweight/obese women, though only the ln(DMA) association was statistically significant.

Maternal urinary arsenic was inversely associated with infant adiposity measured as ponderal index, and the associations appeared limited to women who were overweight or obese prepregnancy. Among children of those women, each doubling of tAs was associated with a 0.55-kg/m^3^ lower (95% CI: –0.82, –0.28, *p* < 0.001) ponderal index. Additionally among those women, decreases in mean ponderal index also were observed with increases in urinary iAs, MMA, and DMA. In contrast, there were not any statistically significant associations between maternal arsenic concentrations and infant ponderal index among normal weight women.

Maternal urinary arsenic was not significantly associated with gestational age, absolute birth weight (Table 2), or birth weight percentiles (see Table S2). The PMI and SMI were not significantly associated with gestational age or any birth anthropometric measures (data not shown).

In analyses that examined effect modification by infant sex (see Table S3), arsenic exposure was positively and significantly related to birth length in male infants but showed no association in females [*p* for interaction for ln(tAs) = 0.04]; among male infants, each doubling of tAs was associated with a 0.28-cm increase (95% CI: 0.09, 0.46, *p* < 0.01) in birth length. Results were similar for DMA and MMA. There was also a suggestive interaction between arsenic exposure and infant sex on the birth weight outcome; arsenic appeared negatively associated with birth weight in girls and positively associated with birth weight in boys, though none of the point estimates were statistically significant, nor were any of the interaction tests.

In analyses stratified by both maternal weight status and infant sex (Table 3), the positive association between arsenic exposure and infant length appeared limited to the stratum of boys born of overweight/obese mothers, though the test for three-way interaction was only marginally significant (*p* = 0.13). In the stratum of boys born of overweight/obese mothers, each doubling of tAs was associated with a 0.51-cm increase (95% CI: 0.21, 0.82) in birth length. When looking at the birth weight outcome, there was a strong, statistically significant negative association between arsenic exposure and birth weight in the stratum of girls born of overweight/obese mothers, whereas associations in the other strata were largely positive and not statistically significant; the test for three-way interaction was significant (*p* = 0.03). In the stratum of girls born of overweight/obese mothers, each doubling of tAs was associated with a 62.9-g decrease (95% CI: –111.6, –14.2) in birth weight.

**Table 3:** Adjusted parameter estimates (95% CI) for the change in birth outcomes with each unit increase in natural log–transformed maternal 2nd-trimester urinary arsenic by maternal prepregnancy weight status*^a^* and infant sex strata.*^b^*

Maternal prepregnancy weight status/infant sex	Arsenic exposure	β (95% CI)		
		Birth length (cm)	Birth weight (g)	Ponderal index (kg/m^3^)
Normal weight				
Female (*n* = 204)	Ln(iAs)	–0.04 (–0.36, 0.27)	–7.1 (–59.3, 45.2)	0.00 (–0.40, 0.40)
	Ln(MMA)	0.02 (–0.24, 0.29)	14.9 (–29.2, 58.9)	0.05 (–0.29, 0.39)
	Ln(DMA)	0.03 (–0.36, 0.42)	34.5 (–29.2, 98.3)	0.17 (–0.32, 0.66)
	Ln(tAs)	0.00 (–0.40, 0.40)	23.5 (–42.4, 89.4)	0.13 (–0.38, 0.63)
Male (*n* = 202)	Ln(iAs)	0.04 (–0.24, 0.32)	–0.7 (–50.9, 49.4)	–0.04 (–0.45, 0.36)
	Ln(MMA)	0.04 (–0.20, 0.27)	20.5 (–22.0, 63.0)	0.12 (–0.23, 0.46)
	Ln(DMA)	0.17 (–0.16, 0.49)	11.6 (–45.8, 69.0)	–0.12 (–0.58, 0.34)
	Ln(tAs)	0.13 (–0.20, 0.47)	3.7 (–56.1, 63.4)	–0.14 (–0.62, 0.34)
Overweight/obese
Female (*n* = 153)	Ln(iAs)	0.00 (–0.32, 0.32)	–58.6 (–115.2, –1.9)	–0.50 (–0.97, –0.03)
	Ln(MMA)	–0.07 (–0.38, 0.25)	–85.0 (–139.2, –30.8)**	–0.58 (–1.04, –0.12)
	Ln(DMA)	0.00 (–0.41, 0.40)	–90.7 (–160.6, –20.8)*	–0.69 (–1.27, –0.10)
	Ln(tAs)	0.01 (–0.40, 0.41)	–90.7 (–161.0, –20.5)*	–0.73 (–1.32, –0.14)
Male (*n* = 147)	Ln(iAs)	0.34 (–0.09, 0.77)	16.9 (–65.2, 98.9)	–0.41 (–0.96, 0.15)
	Ln(MMA)	0.45 (0.14, 0.76)**	28.6 (–31.2, 88.4)	–0.50 (–0.90, –0.09)
	Ln(DMA)	0.75 (0.32, 1.17)**	39.2 (–44.5, 122.9)	–0.95 (–1.50, –0.39)
	Ln(tAs)	0.74 (0.30, 1.19)**	38.7 (–47.7, 125.0)	–0.94 (–1.51, –0.36)
*p* for 3-way interaction between infant sex, maternal weight status and Ln(tAs)^*c*^		0.13	0.03	0.89
^***a***^Normal weight defined as prepregnancy BMI 18.5–24.9 kg/m^2^ and overweight/obese defined as prepregnancy BMI ≥ 25 kg/m^2^; 25 underweight mothers (BMI < 18.5 kg/m^2^) excluded from analysis. ^***b***^Results from a series of regression models fitting each birth outcome on each arsenic exposure measure [natural log–transformed inorganic arsenic (iAs), monomethylarsonic acid (MMA), dimethylarsinic acid (DMA), total arsenic (iAs + MMA + DMA) (tAs)]. All models adjusted for maternal age (continuous), education level (ordinal with the five categories listed in Table 1 assigned values of 1–5, respectively), parity (continuous), and gestational age (continuous). Models adjusted for maternal age (continuous), education level (ordinal with the five categories listed in Table 1 assigned values of 1–5, respectively), parity (continuous), gestational age (continuous) and infant sex. ^***c***^To test for a 3-way interaction between maternal weight status, infant sex, and Ln(tAs), regressions were fit as described in footnote *b* with the addition of six 2-way multiplicative interaction terms between maternal weight status, infant sex, and arsenic exposure, and one 3-way multiplicative interaction term between all 3 variables; *p-*value from a two-sided Wald *t*-test on the coefficient for the 3-way multiplicative term. Results for test of interaction with Ln(MMA) and Ln(DMA) are similar (data not shown). *p < 0.05. **p < 0.01.				

Results were consistent in analyses limited to the subset of women who did not consume seafood (*n* = 594) or rice (*n* = 543) in the previous 2 days, with additional adjustment for creatinine (data available for 520 participants; data not shown), or with additional adjustment for gestational weight gain (data available for 639 participants; data not shown). Also, we did not find evidence that the results of the maternal glucose challenge test or maternal hyperglycemia status modified the association between maternal urinary tAs and infant ponderal index (data not shown).

## Discussion

In this U.S. sample of women and their newborn infants, we found that second-trimester maternal urinary arsenic concentration, a measure of fetal *in utero* arsenic exposure, was negatively associated with infant head circumference, positively associated with infant length and, negatively associated with adiposity at birth. We further observed that the associations between arsenic exposure and birth length were observed only in males, and the associations with adiposity were only observed in the overweight/obese stratum and not in the normal weight stratum. Finally, we observed a significant three-way interaction between maternal weight status, infant sex, and arsenic exposure on the birth weight outcome; specifically, arsenic exposure is related to lower birth weight in females born to overweight/obese mothers, but the association appears null, or possibly in the opposite direction, for all males and for females born of healthy weight mothers.

Our findings relating arsenic exposure and head circumference at birth agree with those from a large prospective analysis by Rahman et al. (2009). In that study of 805 Bangladeshi mothers with urinary arsenic in the lower range of exposure (< 100 μg/L) and their children, 1 μg/L of maternal tAs (average from samples taken around gestational weeks 8 and 30) was associated with a 0.05-mm (SE = 0.03, *p* = 0.04) lower newborn head circumference after adjustment for maternal BMI and socioeconomic status. An analysis of ultrasound measurements from the Bangladeshi cohort (*n* = 1,929) found that arsenic may influence head circumference before the third trimester and in a sex-specific manner (Kippler et al. 2012); maternal urinary arsenic was negatively associated with ultrasound measures of head circumference in males and positively associated with head circumference in females. Our previous analysis of 223 ultrasound reports from our cohort also suggests that this arsenic-related reduced head circumference may be present as early as the second trimester, though we observed a stronger negative association in female fetuses (Davis et al. 2015). In the present study, we did not observe any significant effect modification of the associations between arsenic and head circumference by infant sex.

In contrast to our results, Rahman et al. (2009) did not observe an association between arsenic exposure and birth length. They did observe a negative association between maternal urinary arsenic and birth weight; each 1 μg/L of urinary tAs was associated with an adjusted –1.68 g (SE = 0.62, *p* < 0.01) difference in birth weight when examining infants born of mothers with urinary arsenic concentrations < 100 μg/L. In contrast, we only observed a negative association between arsenic and birth weight in girls born of overweight/obese mothers.

Three other prospective studies have examined maternal urinary arsenic in relation to birth outcomes (Chou et al. 2014; Laine et al. 2015; Shirai et al. 2010). A study of 299 mother–child pairs from a non–arsenic-endemic region of Taiwan (Chou et al. 2014) and a study of 78 low-exposure mother–child pairs from Tokyo (Shirai et al. 2010), did not find any associations between maternal urinary arsenic and head circumference, birth length, or birth weight. In a study of 200 mother–child pairs from an arsenic-endemic region in Mexico, maternal urinary MMA was negatively associated with birth weight and gestational age, and iAs was negatively associated with birth length and gestational age (Laine et al. 2015). In a small study that compared 19 mothers from a city with high water arsenic to 19 mothers from a city with low water arsenic in Chile, there were no significant urinary arsenic–birth weight associations in (Hopenhayn et al. 2003).

The results are also mixed from other studies that relied on maternal hair (Huyck et al. 2007), maternal blood (Claus Henn et al. 2016; Guan et al. 2012), infant meconium (Vall et al. 2012), and drinking-water (Hopenhayn et al. 2003; Kwok et al. 2006; Myers et al. 2010; Yang et al. 2003) arsenic levels to examine the association between arsenic and birth weight (Claus Henn et al. 2016; Huyck et al. 2007; Guan et al. 2012; Hopenhayn et al. 2003; Kwok et al. 2006; Myers et al. 2010; Vall et al. 2012; Yang et al. 2003); length (Claus Henn et al. 2016; Guan et al. 2012; Vall et al. 2012); and head circumference (Claus Henn et al. 2016; Guan et al. 2012; Vall et al. 2012).

Several population characteristics could lead to the heterogeneity of study findings. Our research differs from the bulk of previous research on arsenic and fetal growth because of the relatively low levels of arsenic exposure in our population; for example, participants in the study by Rahman et al. (2009) had a median concentration of 95 μg/L of total urinary arsenic compared with our median of 3.4 μg/L. In addition, almost half of our study population was overweight or obese. In contrast, only 5% of the mothers in the Rahman et al. (2009) study were overweight or obese and 30% were underweight. We were unable to explore the relation between arsenic and fetal growth in underweight women because only 25 women in our sample met this criterion. In addition to weight status, other nutritional factors such as folate (Gamble et al. 2007; Hall et al. 2009) and protein intake (Heck et al. 2009; Milton et al. 2004) could modify the associations between arsenic and fetal growth; thus nutritional factors may lead to heterogeneity in study findings and should be explored in future research.

Fetal growth is a complicated process that is affected by several factors including maternal nutrition, genetics, and toxic exposures (Kramer 1987). Although the mechanism by which arsenic might affect fetal growth is unknown, several possibilities have been proposed. Our previous work suggests that increased maternal arsenic exposure may lead to increased expression of the arsenic transporter AQP9 in placental tissue, which, in turn, may decrease expression of the adipose tissue–derived ENPP2 (Fei et al. 2013), a phospholipase that is purported to regulate adipose tissue growth (Nishimura et al. 2014). In addition, there is evidence that arsenic increases oxidative stress (Xu et al. 2008) and inflammatory processes (Ahmed et al. 2011; Fry et al. 2007), factors associated with growth restriction (Biri et al. 2007; Challis et al. 2009). Given that excess adiposity is also known to increase oxidative stress and systemic inflammation (Vincent and Taylor 2006), it is feasible that arsenic exposure may differentially impact fetal growth depending on maternal weight status. In addition, arsenic could influence fetal growth through other mechanisms such as endocrine disruption (Bodwell et al. 2004; Davey et al. 2007), epigenetic modification (Koestler et al. 2013), and/or altered transcription (Kumagai and Sumi 2007) and it is plausible that effects could differ by infant sex.

Opposite effects of *in utero* arsenic exposure on fetal length at lower and higher exposure doses is plausible given the findings of an *in vitro* study of arsenic trioxide (ATO) and the growth of osteoblasts—the specialized cells in the bone that secrete collagen and a calcium- and phosphate-containing mineral to form the bone matrix (Xu et al. 2014). In that study, a low-dose (0.25, 0.5, and 1 μM) of ATO, an oxide of inorganic arsenic, greatly enhanced the viability of cultured osteoblasts and promoted collagen synthesis and secretion, and a high-dose (5, 10, and 20 μM) of ATO led to a significant reduction in osteoblast viability and induced osteoblast apoptosis. Although we did not observe an inverse association between arsenic and birth length at any part of the dose–response curves in the stratified or total samples, this may be attributable to the relatively low range of arsenic exposure in our population.

Alterations of fetal growth related to *in utero* arsenic exposure may have important public health implications. Head circumference closely correlates to brain volume (Cooke et al. 1977; Wickett et al. 2000), and some research suggests that head circumference at birth is associated with later intellectual function (Gale et al. 2006). Additionally, the persistence of the negative association between arsenic and head circumference after adjusting for birth weight suggests that the reduction in head circumference is independent of birth size, as has been observed with other toxicant exposure (Fein et al. 1984; Hernandez-Avila et al. 2002). Thus, our findings of increased arsenic exposure related to decreased head circumference are consistent with laboratory studies that find that arsenic has neurotoxic effects (Chaudhuri et al. 1999), and epidemiological studies that suggest that arsenic exposure is related to decreased intellectual function (Hamadani et al. 2011; Roy et al. 2011). Other studies suggest that postnatal head growth is also important in determining later cognitive function (Gale et al. 2004; Smithers et al. 2013), so it is important to further explore arsenic’s relationship with postnatal head circumference growth and ultimately cognitive function in future studies.

The influence of fetal growth on later risk of adiposity (Curhan et al. 1996a, 1996b) and metabolic disease (Barker et al. 1989a, 1989b) is well recognized. Studies suggest a U-shaped relationship with both high birth weight/adiposity (Eriksson et al. 2001) and low birth weight/adiposity (Barker et al. 1993; Bhargava et al. 2004) and later adiposity and metabolic disease. Studies additionally suggest that low size at birth may be related to accelerated weight gain (catch up growth) in infancy (Barker et al. 1993; Bhargava et al. 2004) which is in turn related to later obesity (Hack et al. 1984; Ong et al. 2000; Victora et al. 2001). Interestingly, our observed negative association between arsenic exposure and ponderal index in the overweight/obese stratum seems to be driven by longer birth length in boys, but by lower birth weight in girls. Such possible mechanistic differences in adiposity at birth could have important ramifications for later growth trajectories. Ongoing follow-up of our longitudinal cohort will allow us to examine weight and adiposity trajectories in early infancy, and ultimately childhood overweight or obesity status, in relation to *in utero* arsenic exposure in our population. We will also be able to explore whether early childhood arsenic exposure is related to early childhood growth, as has been done in more highly exposed populations (Gardner et al. 2013). A study of 1,505 mother–infant pairs in rural Bangladesh found an inverse relationship between children’s weight, height, and growth velocity and concurrent arsenic exposure at 5 years of age (Gardner et al. 2013).

Unlike the majority of epidemiological studies on arsenic exposure, this population-based prospective study explores arsenic exposures at levels commonly encountered in the United States through water and food sources. Our study is strengthened by using sensitive biomarkers of *in utero* exposure to both inorganic arsenic and its metabolites as well as clinically measured anthropometry at birth. Although we have only a single measure of maternal urinary arsenic during pregnancy, a study in American-Indian communities suggests that urinary arsenic is relatively stable over time (Navas-Acien et al. 2009). Our study was also limited by our use of self-reported prepregnancy weight, which is likely slightly underreported (Holland et al. 2013; Russell et al. 2013); this may have led to some women who were overweight/obese being misclassified as healthy weight. In this analysis, we explored only arsenic species and did not examine arsenic’s correlation with other metals, so we cannot rule out the potential for confounding by those factors. In addition, we did not adjust for micronutrient status, which may relate to arsenic metabolism; however, we believe it is unlikely that this would have substantially influenced our results, beacause all women received prenatal care at study clinics and approximately 92% of women in the cohort report taking prenatal vitamins. A further limitation is that we were underpowered to explore arsenic’s association specifically with risk for being born SGA or LGA, given the small number of infants born with these outcomes.

Our study, in a U.S. cohort of mothers and infants, suggest that arsenic exposure, even at low levels, may affect fetal growth, and that associations may be modified by maternal weight status and infant sex. The health implications of our findings will be elucidated by future longitudinal studies in our cohort and others that explore the impact of prenatal and postnatal arsenic exposure on growth and neurodevelopmental outcomes.

## Supplemental Material

(246 KB) PDF
