# Supplementary material for: Relation between in Utero Arsenic Exposure and Birth Outcomes in a Cohort of Mothers and Their Newborns from New Hampshire
Source: Environ Health Perspect. 2016 Mar 8;124(8):1299–307. doi: 10.1289/ehp.1510065 (PMC4977046; doi:10.1289/ehp.1510065)
Supplement: (246 KB) PDF [file ehp.1510065.s001.acco.pdf]

Environ Health Perspect
DOI: 10.1289/ehp.1510065

Note to readers with disabilities: *EHP* strives to ensure that all journal content is accessible to all readers. However, some figures and Supplemental Material published in *EHP* articles may not conform to 508 standards due to the complexity of the information being presented. If you need assistance accessing journal content, please contact ehp508@niehs.nih.gov. Our staff will work with you to assess and meet your accessibility needs within 3 working days.

Supplemental Material

Relation between *in Utero* Arsenic Exposure and Birth Outcomes in a Cohort of Mothers and Their Newborns from New Hampshire

Diane Gilbert-Diamond, Jennifer A. Emond, Emily R. Baker, Susan A. Korrick, and Margaret R. Karagas

Table of Contents

Table S1. Adjusted parameter estimates (95% confidence interval) for the change in head circumference with each unit increase in natural log transformed maternal 2nd trimester urinary arsenic for all participants and by maternal pre-pregnancy weight status, with additional model adjustment for delivery type or birth weight (N=706)

Table S2. Adjusted parameter estimates (95% confidence interval) for the change in birth length or weight percentile with each unit increase in natural log transformed maternal 2nd trimester urinary arsenic for all participants and by maternal pre-pregnancy weight status (N=706)

Table S3. Adjusted parameter estimates (95% confidence interval) for the change in birth outcomes with each unit increase in natural log transformed maternal 2nd trimester urinary arsenic by infant sex

Table S1. Adjusted parameter estimates (95% confidence interval) for the change in head circumference with each unit increase in natural log transformed maternal 2nd trimester urinary arsenic for all participants and by maternal pre-pregnancy weight status, with additional model adjustment for delivery type or birth weight (N=706).[a,b]

Outcome	Overall (N=706)	Normal Weight (N=406)	Overweight/Obese (N=300)	*P* for interaction[c]
Head Circumference (cm), also adjusted for delivery mode				
Ln(iAs)	-0.09 (-0.20, 0.01)	-0.13 (-0.27, 0.01)	-0.04 (-0.20, 0.12)	0.42
Ln(MMA)	-0.10 (-0.19, -0.01)*	-0.08 (-0.20, 0.04)	-0.12 (-0.26, 0.02)	0.71
Ln(DMA)	-0.12 (-0.24, 0.01)	-0.15 (-0.31, 0.02)	-0.08 (-0.26, 0.11)	0.58
Ln(tAs)	-0.13 (-0.26, -0.01)*	-0.17 (-0.34, 0.00)	-0.09 (-0.28, 0.10)	0.54
Head Circumference (cm), also adjusted for birth weight				
Ln(iAs)	-0.07 (-0.16, 0.03)	-0.11 (-0.24, 0.01)	0.00 (-0.15, 0.15)	0.252
Ln(MMA)	-0.11 (-0.19, -0.03)**	-0.10 (-0.22, 0.01)	-0.11 (-0.23, 0.02)	0.996
Ln(DMA)	-0.13 (-0.24, -0.02)*	-0.16 (-0.32, 0.00)	-0.07 (-0.24, 0.10)	0.394
Ln(tAs)	-0.13 (-0.25, -0.02)*	-0.18 (-0.33, -0.02)*	-0.08 (-0.25, 0.09)	0.411
Head Circumference (cm), also adjusted for delivery mode and birth weight				
Ln(iAs)	-0.07 (-0.17, 0.02)	-0.12 (-0.26, 0.01)	0.00 (-0.12, 0.13)	0.217
Ln(MMA)	-0.11 (-0.19, -0.03)**	-0.11 (-0.22, 0.01)	-0.10 (-0.20, 0.01)	0.819
Ln(DMA)	-0.13 (-0.24, -0.02)*	-0.18 (-0.34, -0.02)*	-0.06 (-0.20, 0.09)	0.242
Ln(tAs)	-0.13 (-0.25, -0.02)*	-0.19 (-0.35, -0.02)*	-0.07 (-0.21, 0.08)	0.260

[a]Normal weight defined as pre-pregnancy BMI 18.5 -24.9 kg/m^2 and overweight/obese defined as pre-pregnancy BMI ≥ 25kg/m^2; 25 underweight mothers (BMI < 18.5 kg/m^2) excluded from analysis.
[b]Results from a series of regression models fitting head circumference on each arsenic exposure measure (natural log transformed inorganic arsenic (iAs), monomethylarsonic acid (MMA), dimethylarsinic acid (DMA), total arsenic (iAs+MMA+DMA) (tAs));

DOI: 10.1289/ehp.1510065

model adjusted for maternal age (continuous), education level (ordinal with the five categories listed in Table 1 assigned values of 1–5, respectively), and parity (continuous); models for overall sample additionally adjusted for maternal pre-pregnancy weight status (categorical).
[c]*P*-value from two-sided Wald's t-test testing the significance of a multiplicative term of maternal pre-pregnancy weight status (categorical) and each arsenic exposure in the regression model.
*$P<0.05$, **$P<0.01$

Table S2. Adjusted parameter estimates (95% confidence interval) for the change in birth length or weight percentile with each unit increase in natural log transformed maternal 2nd trimester urinary arsenic for all participants and by maternal pre-pregnancy weight status (N=706).[a,b]

Outcome/ Maternal Urinary Arsenic Variable	Overall (N=706)	Normal Weight (N=406)	Overweight/Obese (N=300)	*P* for interaction[c]
Birth length percentile				
Ln(iAs)	0.7 (-1.2, 2.6)	0.1 (-2.4, 2.6)	1.5 (-1.5, 4.5)	0.50
Ln(MMA)	1.0 (-0.7, 2.6)	0.4 (-1.8, 2.5)	1.8 (-0.7, 4.4)	0.38
Ln(DMA)	2.1 (-0.1, 4.4)	1.0 (-2.0, 4.0)	3.6 (0.2, 7.0)*	0.26
Ln(tAs)	1.9 (-0.4, 4.2)	0.7 (-2.3, 3.8)	3.5 (0.0, 6.9)	0.24
Birth weight percentile				
Ln(iAs)	-0.3 (-2.0, 1.4)	0.2 (-2, 2.5)	-1.1 (-3.8, 1.6)	0.45
Ln(MMA)	0.3 (-1.2, 1.7)	1.1 (-0.8, 3.0)	-0.9 (-3.1, 1.4)	0.20
Ln(DMA)	0.2 (-1.8, 2.2)	1.3 (-1.3, 4.0)	-1.2 (-4.2, 1.8)	0.22
Ln(tAs)	-0.1 (-2.1, 2.0)	0.9 (-1.8, 3.7)	-1.3 (-4.4, 1.8)	0.29

[a]Normal weight defined as pre-pregnancy BMI 18.5 -24.9 kg/m^2 and overweight/obese defined as pre-pregnancy BMI ≥ $25 kg/m^2$; 25 underweight mothers (BMI < 18.5 kg/m^2) excluded from analysis.
[b]Results from a series of regression models fitting each birth size outcome on each arsenic exposure measure (natural log transformed inorganic arsenic (iAs), monomethylarsonic acid (MMA), dimethylarsinic acid (DMA), total arsenic (iAs+MMA+DMA) (tAs)); model adjusted for maternal age (continuous), education level (ordinal with the five categories listed in Table 1 assigned values of 1–5, respectively), and parity (continuous); models for overall sample additionally adjusted for maternal pre-pregnancy weight status (categorical).
[c]*P*-value from two-sided Wald's t-test testing the significance of a multiplicative term of maternal pre-pregnancy weight status (categorical) and each arsenic exposure in the regression model.
*$P<0.05$

Environ Health Perspect
DOI: 10.1289/ehp.1510065

Table S3. Adjusted parameter estimates (95% confidence interval) for the change in birth outcomes with each unit increase in natural log transformed maternal 2nd trimester urinary arsenic by infant sex.[a]

**Outcome/ Maternal Urinary Arsenic Variable**	**Females (N=357)**	**Males (N=349)**	***P* for interaction**[b]
Gestational Age (weeks)[c]			
Ln(iAs)	0.11 (-0.03, 0.25)	-0.03 (-0.18, 0.12)	0.17
Ln(MMA)	0.10 (-0.03, 0.22)	0.00 (-0.12, 0.12)	0.29
Ln(DMA)	0.13 (-0.05, 0.30)	0.06 (-0.11, 0.22)	0.55
Ln(tAs)	0.15 (-0.03, 0.33)	0.04 (-0.13, 0.21)	0.37
Head Circumference (cm)[d]			
Ln(iAs)	-0.14 (-0.29, 0.00)*	-0.02 (-0.18, 0.13)	0.27
Ln(MMA)	-0.08 (-0.21, 0.05)	-0.13 (-0.25, 0.00)	0.60
Ln(DMA)	-0.08 (-0.26, 0.10)	-0.16 (-0.33, 0.01)	0.52
Ln(tAs)	-0.11 (-0.30, 0.08)	-0.17 (-0.34, 0.01)	0.67
Birth length (cm)[d]			
Ln(iAs)	-0.02 (-0.25, 0.20)	0.15 (-0.08, 0.38)	0.30
Ln(MMA)	-0.02 (-0.22, 0.19)	0.21 (0.03, 0.40)*	0.10
Ln(DMA)	0.00 (-0.27, 0.28)	0.41 (0.16, 0.67)**	0.03
Ln(tAs)	-0.01 (-0.29, 0.28)	0.40 (0.13, 0.66)**	0.04
Birth weight (cm)[d]			
Ln(iAs)	-28.5 (-68.4, 11.38)	10.4 (-30.89, 51.68)	0.19
Ln(MMA)	-22.5 (-58.19, 13.12)	25.9 (-7.31, 59.12)	0.05
Ln(DMA)	-20.5 (-69.49, 28.59)	24.6 (-21.11, 70.34)	0.19
Ln(tAs)	-27.3 (-77.28, 22.66)	21.2 (-26.10, 68.50)	0.17
Ponderal Index[d]			
Ln(iAs)	-0.21 (-0.52, 0.10)	-0.13 (-0.45, 0.18)	0.73

Environ Health Perspect
DOI: 10.1289/ehp.1510065

Ln(MMA)	-0.17 (-0.44, 0.11)	-0.13 (-0.38, 0.13)	0.82
Ln(DMA)	-0.18 (-0.56, 0.20)	-0.46 (-0.81, -0.11)	0.28
Ln(tAs)	-0.23 (-0.61, 0.16)	-0.46 (-0.82, -0.10)	0.39

[a]Results from a series of regression models fitting each birth outcome on each arsenic exposure measure (natural log transformed inorganic arsenic (iAs), monomethylarsonic acid (MMA), dimethylarsinic acid (DMA), total arsenic (iAs+MMA+DMA) (tAs)).
[b]*P*-value from a two-sided Wald's t-test testing the significance of a multiplicative term of infant sex and each arsenic exposure in the regression model.
[c]Models for gestational age adjusted for maternal age (continuous), education level (ordinal with the five categories listed in Table 1 assigned values of 1–5, respectively), parity (continuous), and maternal pre-pregnancy weight status (categorical: BMI 18.5 -24.9 kg/m^2, BMI $\geq$ 25kg/m^2).
[d]Models for head circumference, birth length, birth weight, and Ponderal index outcomes adjusted for variable described in footnote c and also for gestational age (continuous).
*P<0.05; **P<0.01
